# Maternal sleep position: what do we know where do we go?

**DOI:** 10.1186/1471-2393-15-S1-A4

**Published:** 2015-04-15

**Authors:** Louise M O’Brien, Jane Warland

**Affiliations:** 1 Sleep Disorders Center, Department of Neurology and the Department of Obstetrics & Gynecology, University of Michigan, Ann Arbor, Michigan, USA; 2 School of Nursing and Midwifery, University of South Australia, Adelaide South Australia, Australia

## 

Good sleep is an essential component to health and wellbeing. It consumes one third of human existence; unhealthy sleep can severely impair the other two-thirds. An increasing amount of data now shows that poor sleep – such as sleep disordered breathing, poor sleep quality, and insomnia - has a negative impact on pregnancy outcomes [1-5]. Indeed, over half of the most important risk factors for stillbirth, such as maternal hypertension, gestational diabetes, and fetal growth restriction, have been shown to be associated with maternal sleep disruption [1,2,6-9]. Findings from recent studies have also suggested that maternal sleep position may be a risk factor for stillbirth [10,11]. It has long been recognized that posture in late pregnancy can have a profound effect on maternal hemodynamics. Studies in awake pregnant women have demonstrated reduced ejection fraction and cardiac output in the supine position compared to the left lateral position [12] that may reduce utero-placental blood flow to the fetus since the gravid uterus compresses the inferior vena cava. Failure to prevent this compression can lead to maternal supine hypotensive syndrome [13] and to an adverse effect on umbilical artery blood flow and gas exchange between mother and fetus, with consequent fetal heart rate decelerations [14] and fetal growth restriction [15].

For over 60 years it has been standard of care to place laboring pregnant women in the left lateral tilt position to displace the uterus from the inferior vena cava and improve maternal hemodynamics. Despite this knowledge, little attention has been paid to maternal sleep position during pregnancy even though we spend about one third of our life asleep. Given the known effects of inferior vena cava compression it is very possible that supine sleep could be a risk for stillbirth. Recent studies in Auckland, New Zealand [10], and Ghana, Africa [11] have both shown that supine sleep is independently associated with stillbirth; indeed Owusu et al [11] found that the effect of supine sleep on stillbirth was mediated via low birth weight. Both of the latter studies suggested that if supine sleep plays a causal role in stillbirth, altering the sleep position of pregnant women may reduce stillbirth by approximately 25%. Of note, we have recently demonstrated that the majority of pregnant women (about 80%) spend some time sleeping supine, with the median time being approximately one quarter of the night [16].Supine sleep may therefore represent a maternal stressor in the unexplained late stillbirth triple risk model [17]. Thus, if supine sleep plays a role in stillbirth, the majority of pregnant women would benefit from education and potential intervention. Several potential methods to reduce supine sleep include the use of mattress wedges or pillows [18] or other interventions such as the ‘tennis ball’ technique [19] or even novel devices that could alert a pregnant women to change position. However, before intervention studies are launched, it is pertinent that the findings regarding sleep position are repeated and confirmed in other studies; several such studies are currently underway including small studies monitoring the fetus during maternal sleep (O’Brien and Warland, personal communication) and large studies such as the MiNESS study in the UK [20] that will either support or refute the sleep position hypothesis.
